# Supplementary material for: Three weeks of rehabilitation improves walking capacity but not daily physical activity in patients with multiple sclerosis with moderate to severe walking disability
Source: PLoS One. 2022 Sep 19;17(9):e0274348. doi: 10.1371/journal.pone.0274348 (PMC9484681; doi:10.1371/journal.pone.0274348)
Supplement: Attachment — Submitted filename: response to reviewers.docx [file pone.0274348.s003.docx]

30^th^ July, 2022

*Reply to the reviewer*

*Thank you for your positive feedback on our manuscript, and for the extensive suggestions for improvements, which we have copied in this letter. We have noted our changes and comments in italics below, based on your suggestions. We hope that we have addressed all of the comments to your satisfaction.*

*The authors.*

Reviewer #1:

It is interesting to note that the standard components of MS rehabilitation when conducted inpatient for 3 weeks improved walking capacity, mobility, and motor and cognitive fatigue. As expected, the inpatient rehabilitation for 3 weeks did not have an effect on the physical activity levels after discharge, as there were no specific interventions to improve daily physical activity levels post-discharge.

1. I notice that you have adjusted your regression models for disease severity (line 190) (or walking disability severity – line 232), and other potential confounders. Can you pls clarify whether you have used total EDSS scores or any other specific walking disability measure for these analyses?

*We have clarified this in the text, which now reads, “Linear regression models were used to adjust for the confounding effects of walking disability severity, weekends, baseline PA, age, and sex. Walking disability severity was defined as a categorial variable describing either mild/moderate disability (EDSS <= 5) or severe disability (EDSS>5).” (Line 200-203)*

2. Some researchers debate that the difference in disability status between EDSS scores are not equal (for e.g., EDSS 3.0 to 3.5 vs 6.0 to 6.5 represent no vs large change in walking disability). I see that the inter-quartile range for EDSS scores at baseline were 4.5 to 6.5. Does this mean that there were participants with no walking impairment (EDSS 3.0 to 3.5) in your study? If so, I am thinking whether such a limitation of EDSS scale could have had a confounding effect on your analyses. Pls acknowledge this as a limitation, and/or if necessary, pls revise your analyses.

*This study included 3 participants with EDSS less than 4, though most had higher EDSS. We now note the limited distribution of walking impairment of the population (Line 336 ff). However, by considering walking disability severity as a categorical rather than linear variable, we accounted for this nonlinearity to the extent possible in this population during adjustment. Furthermore, EDSS was treated as a descriptive measure rather than an outcome measure in this study, given its limited and nonlinear responsiveness.*

3. It is nice to read that some of your results are in line with the literature, as per your citations in the discussion. Are you able to report a comparison with adequate data/explanation, instead of textual statements (as in lines 256-7)?

*We have reported results of TUG and 2MWT in comparison with our study. (Line 286 ff)*

4. Since this is an exploratory single group non-randomized observational study, pls acknowledge that the effect sizes presented in the manuscript do not ascribe causality, in your limitation section.

*We have added this limitation (line 343-344).*

5. I see that there were 50% females in your sample (table 1). Pls acknowledge this as a limitation as it does not represent male to female ratio of MS prevalence

*We have added this limitation (line 344-346).*

6. In the introduction, you have mentioned that, in your experience, patients with MS report having either more or no change in physical activity levels after rehabilitation. I notice that the lower end of inter-quartile range for physical activity level has decreased from 202 to 169 min/day between T3 and T4 time points (table 2). Have you considered describing the characteristics of those who had a decrease in their physical activity levels after rehabilitation through a sub-group analysis? Or, you may consider presenting the spread of your individual data points (connected/paired pre-post) using figures with/without error bars.

*At the population level, we observed a significant decrease in PA at T4 compared with baseline. However, we did not observe significant differences between baseline and T3, or between T3 and T4. Most participants (18 of 24) exhibited decreases between baseline and T4, and 11 of these participants exhibited decreases greater than 20%. Participants who exhibited a decrease of 20% or more had significantly higher EDSS than those who did not (Median [Q2-Q3]: 4.75 [4-6] vs 6 [6-6.5], p = 0.011). We now report this in the Physical Activity section of the results (line 267-270). In our limitations section, we discuss potential confounding effects which may have contributed to this decrease, including winter weather and MS progression.*

*This study was not sufficiently powered to conduct other sub-analyses. However, we did include a figure (Figure 3, line 261) to illustrate changes in PA at the population level and the individual level over time.*

7. I acknowledge that relevant statistical analyses were completed to support the conclusions. However, there is no mention of how missing data were handled (other than accelerometer data), if any.

*This was stated and reported (line 213-214): “Data were complete for baseline and outcome measurements”.*

Reviewer #2:

Thank you for the opportunity to review this manuscript. I have read it with great interest.
The authors performed a study on the effects of inpatient rehabilitation on physical activity, walking capacity and fatigue in patients with MS.
I have reviewed the well-conducted and well-reported manuscript and would like to make some minor suggestions on how to improve the quality of reporting.

1. Please avoid abbreviations. Especially FSMCc and FSMCm are not explained.

*Thanks for this remark. We have deleted abbreviations in the abstract.*

2. Please avoid p values according to recent recommendations. Rather report 95% CIs. Please refer to doi: 10.1016/j.physio.2021.12.003

*We have now reported 95%CI in the abstract section.*

3. A “question” such as “Why are PwMS less physically active than the general population?” seems uncommon for a scientific report and may be revised. Eg, “PwMS are less physically active than the general population because of xyz”.

*Agree. We reformulated this: “PwMS are less physically active than the general population.” (Line 61)*

4. Please give examples for the “comorbidities that reduced walking ability” (exclusion criterium).

*We have given examples for comorbidities: “such as musculoskeletal or cardiovascular diseases.” (Line 111-112)*

5. I suggest reporting the recruitment and data collection periods in the methods section (if defined a priori before the data collection).

*See line 93-94: “Recruitment was between 1 August 2017 and 31 March 2018.“*

6. Please provide a sample size calculation or justification/rationale.

*See line 91-93 (Methods, design).*

7. Please state the exact values of the “reported minimum for clinically meaningful changes” used in this study and exceeded by the participants.

*Thanks for your comment. We have added the corresponding values (Line 286 ff).*

8. Line 264: What is the meaning of the EDSS values (EDSS 4.0–6.5)? Range, IQR?

*EDSS 4.0–6.5 is the range defined by the authors Ehling et al. representing “moderate to severe” walking disability. We formulated this accordingly (Line 302-303).*

9. Please avoid questions such as in line 268 and 289.

*Corrected.*

10. Please explain why the heterogeneous population of PwMS is a limitation.

*This statement was incorrect, thanks! We reformulated this to: “Another limitation is that most of the patients had moderate to severe disability. The results cannot be generalized to PwMS with mild disability (Line 349-350).*

11. I suggest to discuss the issues with data collection (“Due to technical issues, wear time validation was not available for most recordings in the current study.”) in the limitations section.

*Agree. We have added the following sentence to the limitations: “For technical reasons a non-standard approach to wear time validation, and this may have affected point estimates of time spent in PA. However, we were able to validate our method in a subset of data against traditional wear time validation methods.” (Line 350 ff).*

12. Since this is an exploratie study, generalisability is not the focus and thus, this is not a weakness of the study. The authors may clearly state this and rather discuss future directions for further research and how this study might inform further studies with a bit more passion.

*Relates to comment 6 and 11 (Line 324 ff).*

Reviewer #3:

The purpose of this study is to assess the effect of rehabilitation on daily physical activity and walking ability in multiple sclerosis patients with moderate to severe walking disability. This study, I believe, is an insightful examination that offers light on an essential problem. However, the author must address a number of critical issues before publication. I have compiled the following list of criticisms that the authors need to address:

1. Why were some exercises given 5 times per week while others 3-5 or 2-3 times/week?

*These varying therapy frequencies represent individualised therapy programs that are determined by rehabilitation goals and available resources. We have clarified this in the methods section (Line 124-125).*

2. How physiotherapy and strength/endurance training are different? What type of interventions were given in physiotherapy? please clarify

*We have specified that strength training was 3 times per week, and endurance training 2 times per week. These treatment frequencies are appropriate for the purpose.*

3. What are the activities included under Neuropsychological training?

*The activities during Neuropsychological training depend on the individual neurocognitive profile and on the rehabilitation goals and aim at improving deficits and learning strategies to compensate for deficits. This is now clarified in the text. We believe that providing a list of activities would be beyond the scope of this paper (Line 129 ff).*

4. Did you assess the reliability of each outcome measure used in the current study?

*In the methods section we refer to the evaluation of reliability of the TUG and 2MWT by Valet et al. The evaluation of reliability of the included outcome measures was not part of the present study.*

5. Did you find any variations in test-retest reliability of any outcome measure?

*We did not evaluate test-retest reliability. Time between repeated outcome measures was too long for this purpose.*

6. Please provide details of sample size estimation.

*……see Reviewer #2 Comment 6……*

7. Follow-up periods were inconsistent. For example, in the beginning it was mentioned 3 months, however, in page 11 it was 2 months? please clarify

*Thanks for this remark. We have corrected this (Line 219).*

8. Please include some figures showing interventions and findings for better understanding and interpretation.

*We have now included Fig 2 (showing interventions) in the result section (line 234). Findings are represented in table 2.*
